# Epidemiologische Daten und medizinische Versorgungssituation von Patienten mit chronischen Entzündungserkrankungen in Deutschland: Real-World-Evidenz zu Prävalenz, Erkrankungskombinationen, Versorgung

**DOI:** 10.1007/s00393-023-01459-7

**Published:** 2023-12-09

**Authors:** Gabriela Riemekasten, Renate Schmelz, Knut Schäkel, Diamant Thaci, Stefan Schreiber, Marit Röcken, Holger Bartz, Tina Ploner, Ximing Liao, Valeria Weber, Karina C. Manz, Harald Burkhardt, Jan Leipe

**Affiliations:** 1 Klinik für Rheumatologie und klinische Immunologie, Universitätsklinikum Schleswig-Holstein, Lübeck, Deutschland; 2 Medizinische Klinik und Poliklinik I, Universitätsklinikum Carl Gustav Carus Dresden, Fetscherstr. 74, 01307 Dresden, Deutschland; 3 Hautklinik, IZEH – Interdisziplinäres Zentrum für chronisch entzündliche Erkrankungen, Universitätsklinikum Heidelberg, Heidelberg, Deutschland; 4 Institut und Exzellenzzentrum für Entzündungsmedizin, Universität zu Lübeck, Lübeck, Deutschland; 5 Klinik für Innere Medizin I und Institut für Klinische Molekularbiologie, Universitätsklinikum Schleswig-Holstein, Campus Kiel, Kiel, Deutschland; 6 Janssen-Cilag GmbH, Neuss, Deutschland; 7 InGef – Institut für angewandte Gesundheitsforschung Berlin GmbH, Berlin, Deutschland; 8 IGES Institut GmbH, Berlin, Deutschland; 9 Medizinische Klinik II/Rheumatologie, Fraunhofer Institut für Translationale Medizin und Pharmakologie (ITMP), Universitätsklinikum Frankfurt, Goethe-Universität Frankfurt am Main, Frankfurt, Deutschland; 10 Sektion Rheumatologie, V. Medizinische Klinik, Universitätsklinikum Mannheim, Mannheim, Deutschland

**Keywords:** Chronisch entzündliche Erkrankungen, Abrechnungsdaten der GKV, Retrospektive Querschnittanalyse, Multidisziplinärer Ansatz, Effizienz der Versorgung, Immune-mediated inflammatory diseases, Health insurances accounting data, Retrospective cross-sectional analysis, Multidisciplinary approach, Efficiency of care

## Abstract

**Hintergrund:**

Chronisch entzündliche Erkrankungen („immune-mediated inflammatory diseases“ [IMID]) können aufgrund klinischer Gemeinsamkeiten überlappen oder gleichzeitig auftreten. Die daraus resultierende Inanspruchnahme von Versorgungsstrukturen wurde bisher nicht fachübergreifend untersucht, ist aber für eine optimierte Behandlung der Patienten mit IMID potenziell von Bedeutung.

**Ziel der Arbeit:**

Analyse epidemiologischer Daten einschließlich Inanspruchnahme von Versorgungsleistungen bei Patienten mit ausgewählten IMID: Psoriasis, Psoriasisarthritis (PsA), rheumatoide Arthritis (RA), Spondylitis ankylosans, Colitis ulcerosa, Morbus Crohn und Kollagenosen.

**Material und Methoden:**

In einer retrospektiven Querschnittanalyse, basierend auf Abrechnungsdaten der gesetzlichen Krankenversicherung (GKV) mit einer Stichprobe von ca. 4 Mio. Versicherten, wurden die Prävalenz o. g. IMID und die Häufigkeit von IMID-Kombinationen anhand dokumentierter Diagnosen (ICD-10 GM) analysiert. Die Häufigkeit von Hospitalisierungen und Inanspruchnahmen ambulanter Arztkontakte wurde in vordefinierten Fachdisziplinen (Allgemeinmedizin, Dermatologie, Gastroenterologie, Rheumatologie) erfasst und mit einer alters- und geschlechtsadjustierten Referenzpopulation verglichen.

**Ergebnisse:**

Insgesamt wiesen 188.440 Patienten mindestens eine der analysierten IMID-Diagnosen auf (4,7 %), mit einem Altersgipfel von 61 bis 70Jahren. Die höchste Prävalenz wurde für die Psoriasis (1,85 %), gefolgt von der rheumatoiden Arthritis (1,38 %) gesehen. Kombinationen mit mindestens einer weiteren IMID kamen insgesamt relativ häufig vor (29 %), wobei dies bei Patienten mit Psoriasisarthritis am häufigsten zu verzeichnen war (82,9 %, wobei hiervon 68,2 % Psoriasis), gefolgt von Spondylitis ankylosans (27,5 %) und Morbus Crohn (21,6 %). Patienten mit IMID wurden im Vergleich zur Referenzpopulation häufiger hospitalisiert und wiesen häufigere Inanspruchnahmen der betrachteten ambulanten Fachdisziplinen auf.

**Diskussion:**

Die Studienergebnisse beschreiben, dass IMIDs gehäuft koexistieren und die Patienten Versorgungsstrukturen verschiedener Fachgebiete vergleichsweise mehr in Anspruch nehmen. Ein multidisziplinärer Ansatz könnte die Effizienz der Versorgung steigern, eine Evaluierung steht aus.

**Zusatzmaterial online:**

Die Online-Version dieses Beitrags (10.1007/s00393-023-01459-7) enthält die Tab. S1–S9 und die STROBE-Checkliste.

## Abstract (en)

**Background:**

Chronic inflammatory diseases (immune-mediated inflammatory diseases, IMID) can overlap or occur simultaneously due to clinical similarities. The resulting utilization of heathcare structures has not yet been investigated across disciplines but is of potential importance for optimizing the treatment of patients with IMID.

**Aim of the work:**

Analysis of epidemiological data including utilization of care services in patients with selected IMIDs: psoriasis, psoriatic arthritis (PsA), rheumatoid arthritis (RA), ankylosing spondylitis, ulcerative colitis, Crohn’s disease and connective tissue disease.

**Material and methods:**

In a retrospective cross-sectional analysis based on health insurances accounting data with a sample of approximately 4 million insured persons, the prevalence of the abovementioned IMID and the frequency of IMID combinations were analyzed based on documented diagnoses (ICD-10 GM). The frequency of hospitalizations and utilization of outpatient physician contacts was recorded in predefined specialist disciplines (general medicine, dermatology, gastroenterology, rheumatology) and compared with an age-adjusted and gender-adjusted reference population.

**Results:**

A total of 188,440 patients had at least 1 of the IMID diagnoses analyzed (4.7%), with an age peak of 61–70 years. The highest prevalence was observed for psoriasis (1.85%), followed by rheumatoid arthritis (1.38%). Combinations with at least one other IMID were relatively common (29%), with this being most common in patients with psoriatic arthritis (82.9%, of which 68.2% had psoriasis), followed by ankylosing spondylitis (27.5%) and Crohn’s disease (21.6%). Compared to the reference population, patients with IMID were hospitalized more often and more frequently utilized the outpatient disciplines.

**Discussion:**

The study results describe that IMIDs occur in combination and that the patients make comparatively more use of care structures of different disciplines. A multidisciplinary approach could increase the efficiency of care; an evaluation is still pending.

## Hintergrund und Fragestellung

Chronisch entzündliche Erkrankungen wie Psoriasis, Psoriasisarthritis (PsA), rheumatoide Arthritis (RA), Spondylitis ankylosans, Colitis ulcerosa, Morbus Crohn und Kollagenosen gehören zu den häufigsten sog. „immune-mediated inflammatory diseases“ (IMID), d. h. immunvermittelten Systemerkrankungen mit komplexer, multifaktorieller Ätiologie, die im Zusammenhang mit einer dysregulierten Immunantwort häufig zu Endorganschäden führen [16]. Diese Krankheitsentitäten unterscheiden sich zwar untereinander v. a. durch die primär betroffenen Organe (z. B. Haut, Gelenke, Darm), weisen aber immunologische, ätiologische und klinische Gemeinsamkeiten auf und betreffen häufig mehrere Organsysteme [5, 26, 27]. Epidemiologisch spiegeln sich diese gemeinsamen pathophysiologischen Mechanismen in einem erhöhten Risiko für die Entwicklung weiterer IMID wider [5, 7, 8, 16, 18, 23]. In einer retrospektiven Kohortenstudie auf der Basis von US-amerikanischen Krankenkassendaten zeigte sich bei Vorliegen einer IMID ein erhöhtes Risiko für die Entwicklung einer weiteren IMID mit Hazard Ratios von 7,5 bei Patienten mit chronisch entzündlichen Darmerkrankungen (CED), 16,8 bei Patienten mit rheumatoider Arthritis (RA) und 62,2 bei Patienten mit Psoriasisarthritis (PsA) [5].

Aufgrund der Überschneidungen hinsichtlich immunologischer und klinischer Charakteristika wird für Patienten mit mehreren IMID zur optimalen Einordnung und Therapiesteuerung ein interdisziplinäres Management empfohlen, u. a. durch eine enge Zusammenarbeit verschiedener Fachdisziplinen wie Dermatologen, Rheumatologen und Gastroenterologen (im Allgemeinen und neuerdings speziell in Entzündungsboards) in Kooperation mit Allgemeinmedizinern [14, 17]. Disziplinübergreifende Ansätze zur Untersuchung der Epidemiologie und Versorgungssituation sind für dieses breite Krankheitsspektrum in der Literatur bisher jedoch kaum vorhanden und eine ganzheitliche Betrachtung der IMID ist selten. So konzentrieren sich bisherige Studien in Deutschland primär auf einzelne Krankheitsentitäten hinsichtlich Epidemiologie [2, 20–22, 28, 30] und Versorgungssituation [11, 12, 29].

## Ziel der Arbeit

Ziel der vorliegenden Studie war es daher, die epidemiologischen Aspekte häufiger IMID der Entzündungsmedizin und deren disziplinübergreifende Versorgungssituation in Deutschland anhand von Abrechnungsdaten der gesetzlichen Krankenversicherung (GKV) zu untersuchen und Rückschlüsse auf den interdisziplinären Versorgungsbedarf ziehen zu können.

## Studiendesign und Untersuchungsmethoden

### Datenbasis und Studiendesign

Die Studie basiert auf der Datenbank des Instituts für angewandte Gesundheitsforschung Berlin (InGef), welche aggregierte, anonymisierte GKV-Längsschnittdaten von rund 60 Krankenkassen und rund 8 Mio. Versicherten in Deutschland umfasst, u. a. auch zu stationären Diagnose- und Leistungsdaten sowie Diagnosen (deutsche Version der Internationalen statistischen Klassifikation der Krankheiten und verwandter Gesundheitsprobleme in der 10. Revision [ICD-10-GM]). Diese retrospektive Querschnittanalyse erfolgte dabei auf Basis einer für die deutsche Bevölkerung hinsichtlich Alter und Geschlecht repräsentativen Stichprobe von rund 4 Mio. Versicherten [6] im Kalenderjahr 2018 als Beobachtungszeitraum.

### Identifikation der Studienpopulation und Bestimmung der Prävalenz

Die Studienpopulation umfasste prävalente Patienten mit mindestens 2 gesicherten ambulanten IMID-Diagnosen in 2 Quartalen (M2Q) oder mit mindestens einer stationären Haupt- oder Nebendiagnose im Studienjahr, wobei Versicherte im Beobachtungszeitraum durchgehend oder bis zum Tod versichert sein mussten. Die 7 von Experten verschiedener Entzündungszentren (Dermatologie, Gastroenterologie und Rheumatologie) in Zusammenarbeit mit InGef und IGES ausgewählten IMID-Entitäten waren: Psoriasis (L40.- ohne L40.5), PsA (L40.5 oder M07.-), RA (M05.- oder M06.-), Spondylitis ankylosans (M45.- und M46.-), Colitis ulcerosa (K51.-), Morbus Crohn (K50.-) und Kollagenosen. Die Gruppe der Kollagenosen umfasste systemischen Lupus erythematodes (M32.-), systemische Sklerose (M34.-), Dermatomyositis/Polymyositis (M33.-) und andere Erkrankungen mit systemischer Beteiligung des Bindegewebes (z. B. M35.- ohne M35.3) (Tabelle S1). Die definierten Krankheitsentitäten wurden sowohl einzeln als auch als aggregierte Gesamtgruppe analysiert, und es wurden 12-Monats-Prävalenzen ermittelt.

Für jede prävalente Patientenpopulation (d. h. jede IMID) wurde eine Referenzpopulation durch ein direktes 1:1-Matching ausgewählt: Jede Referenzpopulation umfasste 1 übereinstimmendes Individuum für jede Person der jeweiligen prävalenten Patientenpopulation. Diese „Zwillinge“ wurden nach dem Zufallsprinzip aus der Datenbank ausgewählt, mit Ausnahme der folgenden Bedingungen: gleiches Alter, gleiches Geschlecht, keine Diagnose für die jeweilige IMID. Darüber hinaus mussten die Personen aus den Referenzpopulationen im Kalenderjahr 2018 durchgehend versichert sein.

### IMID-Kombinationen

Innerhalb der Studienpopulation wurden Kombinationen (Dokumentation mehrerer IMID pro Kalenderjahr [M2Q]) ermittelt, wobei dies als Dokumentation einer weiteren IMID-Diagnose innerhalb des Studienzeitraums nach oben genannten Kriterien definiert wurde.

### Hospitalisierungen und (Fach‑)Arztkontakte

Für die Studienpopulation sowie für die einzelnen IMID-Entitäten wurden die absolute und relative Häufigkeit der Inanspruchnahme von stationären und ambulanten Versorgungsleistungen erfasst. Ebenso wurde die mittlere Anzahl der Arztkontakte pro Patient ermittelt und jeweils mit einer korrespondierenden alters- und geschlechtsadjustierten Referenzpopulation ohne IMID verglichen. Berücksichtigt wurden vollstationäre, teilstationäre und vorstationäre Aufenthalte sowie ambulante Arztkontakte in den Fachdisziplinen Allgemeinmedizin, Dermatologie, Rheumatologie und Gastroenterologie.

## Ergebnisse

**Abb. 1:**
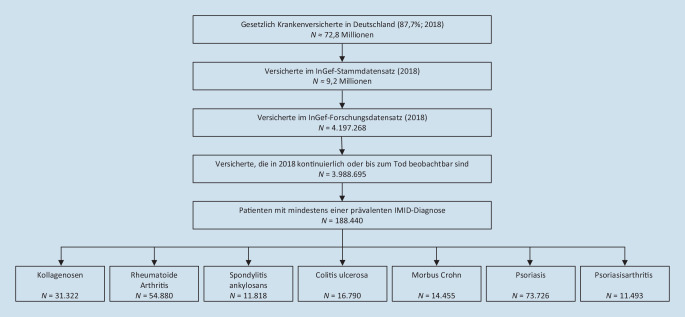
Schritte der Patientenselektion

**Abb. 2:**
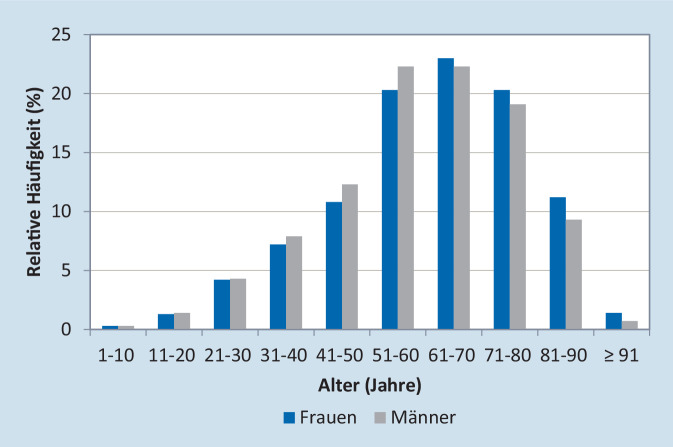
Alters- und Geschlechtsverteilung von prävalenten Patienten mit chronisch entzündlichen Erkrankungen (IMID)

Im Kalenderjahr 2018 betrug die Anzahl der gesetzlich Versicherten in der zugrunde liegenden Stichprobe 4.197.268. Davon waren 3.988.695 durchgehend oder bis zum Tod versichert; 188.440 Patienten wiesen mindestens eine der betrachteten IMID-Diagnosen auf (4,7 %) (Abb. 1). Patienten mit IMID waren zu 57,6 % weiblich und im Durchschnitt 60,8 (± 17,0) Jahre alt. Die Mehrheit der Patienten war zwischen 51 und 80 Jahren alt mit einem Altersgipfel zwischen 61 und 70 Jahren (Abb. 2).

### 12-Monats-Prävalenz für IMID im Jahr 2018

**Abb. 3:**
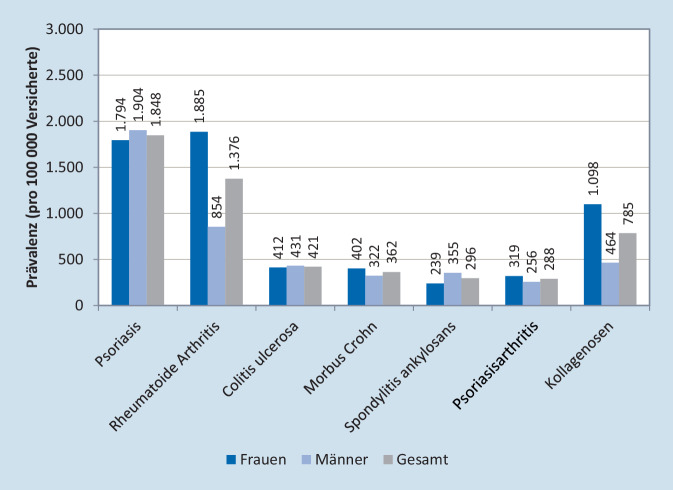
12-Monats-Prävalenz der einzelnen IMID, stratifiziert nach Geschlecht

Die 12-Monats-Prävalenz für alle 7 IMID lag bei 4724 Patientinnen und Patienten pro 100.000 Versicherte (95 %-Konfidenzintervall (CI): 4704–4745) und war bei Frauen höher als bei Männern (5369 vs. 4063 pro 100.000). Innerhalb der einzelnen Krankheitsentitäten war die Prävalenz für Psoriasis mit 1848 pro 100.000 (95 %-CI: 1835–1862) am höchsten, gefolgt von rheumatoider Arthritis (1376 pro 100.000, 95 %-CI: 1365–1387), Kollagenosen (785 pro 100.000, 95 %-CI: 777–794), Colitis ulcerosa (421 pro 100.000, 95 %-CI: 415–427), Morbus Crohn (362 pro 100.000, 95 %-CI: 357–368), Spondylitis ankylosans (296 pro 100.000, 95 %-CI: 291–301) und PsA (288 pro 100.000, 95 %-CI: 283–293) (Abb. 3). Die Prävalenzen der einzelnen IMID, stratifiziert nach Altersgruppen und Geschlecht, sind zudem in den Tabellen S2–S9 dargestellt.

### Auftreten von IMID-Kombinationen im Jahr 2018

Für die betrachteten Entitäten betrug die Häufigkeit mindestens einer weiteren IMID im Untersuchungszeitraum in aufsteigender Reihenfolge bei Psoriasis 16,9 %, RA 20,1 %, Kollagenosen 17,3 %, Colitis ulcerosa 19,3 %, Morbus Crohn 21,6 %, Spondylitis ankylosans 27,5 % und PsA 82,9 % (wobei hiervon 68,2 % Psoriasis hatten, was in der Regel an die Diagnose gebunden ist) der Patienten (Tab. 1). Die häufigsten zusätzlichen IMID waren bei Patienten mit Psoriasis eine PsA (10,6 %), bei RA eine Psoriasis (7,9 %), bei Kollagenosen eine RA (11,5 %), bei Colitis ulcerosa ein Morbus Crohn (10,1 %), bei Morbus Crohn eine Colitis ulcerosa (11,7 %), bei Spondylitis ankylosans eine RA (15,5 %) und bei PsA eine Psoriasis (68,2 %). Patienten aus dem Spondyloarthritis-Komplex, mit Spondylitis ankylosans und PsA, wiesen CED am häufigsten als zusätzliche IMID-Diagnosen auf: Colitis ulcerosa bei 2,2 und 2,6 %; Morbus Crohn bei 2,9 und 3,1 %.

**Tab. 1:** Kombiniertes Auftreten von IMID

					IMID													
	Ohne Auftreten weiterer IMID		Mit Auftreten weiterer IMID		Psoriasis		Rheumatoide Arthritis		Kollagenosen		Colitis ulcerosa		Morbus Crohn		Spondylitis ankylosans		Psoriasisarthritis	
	*n*	%	*n*	%	*n*	%	*n*	%	*N*	%	*n*	%	*n*	%	*n*	%	*N*	%
Psoriasis (*n* = 73.726)	61.262	83,1	12.464	16,9	/	/	4362	5,9	1357	1,8	672	0,9	596	0,8	906	1,2	7833	10,6
Rheumatoide Arthritis (*n* = 54.880)	43.863	79,9	11.017	20,1	4362	7,9	/	/	3607	6,6	649	1,2	565	1,0	1836	3,3	3247	5,9
Kollagenosen (*n* = 31.322)	25.906	82,7	5416	17,3	1357	4,3	3607	11,5	/	/	333	1,1	250	0,8	394	1,3	411	1,3
Colitis ulcerosa (*n* = 16.790)	13.545	80,7	3245	19,3	672	4,0	649	3,9	333	2,0	/	/	1694	10,1	264	1,6	295	1,8
Morbus Crohn (*n* = 14.455)	11.339	78,4	3116	21,6	596	4,1	565	3,9	250	1,7	1694	11,7	/	/	343	2,4	353	2,4
Spondylitis ankylosans (*n* = 11.818)	8563	72,5	3255	27,5	906	7,7	1836	15,5	394	3,3	264	2,2	343	2,9	/	/	567	4,8
Psoriasisarthritis (*n* = 11.493)	1962	17,1	9531	82,9	7833	68,2	3247	28,3	411	3,6	295	2,6	353	3,1	567	4,9	/	/

*IMID* chronische Entzündungserkrankung(en)

### Häufigkeit von Hospitalisierungen

Mit einem Anteil von 28,6 % mussten IMID-Patienten rund 1,5-mal häufiger mindestens 1‑mal stationär behandelt werden als 19,5 % der Vergleichspopulation (Tab. 2). Je nach Krankheitsentität variierte der Anteil mit Hospitalisierung, wobei dieser bei Patienten mit RA (34,7 %) am höchsten war, gefolgt von Morbus Crohn (32,3 %), Kollagenosen (31,0 %), PsA (29,0 %), Colitis ulcerosa (28,5 %) und Spondylitis ankylosans (28,0 %) und Psoriasis (24,4 %).

**Tab. 2:** Hospitalisierungen prävalenter Patienten mit IMID pro Krankheitsentität und jeweiliger Referenzpopulation

	Patienten mit IMID						Referenzpopulation					
	Individuen mit Hospitalisierung		Anzahl Hospitalisierung pro Inanspruchnehmer				Individuen mit Hospitalisierung		Anzahl Hospitalisierung pro Inanspruchnehmer			
	*n*	%	Mittelwert	SD	Median	IQR	*n*	%	Mittelwert	SD	Median	IQR
IMID gesamt (*n* = 188.440)	53.945	28,6	1,9	2,5	1	1–2	36.775	19,5	2,1	4,7	1	1–2
Morbus Crohn (*n* = 14.455)	4667	32,3	1,9	1,8	1	1–2	2157	14,9	1,7	2,2	1	1–2
Colitis ulcerosa (*n* = 16.790)	4784	28,5	1,9	1,7	1	1–2	2889	17,2	2,1	4,4	1	1–2
Psoriasis (*n* = 73.726)	17.984	24,4	1,8	1,6	1	1–2	13.812	18,7	2,0	3,7	1	1–2
Psoriasisarthritis (*n* = 11.493)	3328	29,0	1,8	1,5	1	1–2	1962	17,1	1,9	2,5	1	1–2
Spondylitis ankylosans (*n* = 11.818)	3307	28,0	1,8	1,6	1	1–2	2132	18,0	1,9	2,6	1	1–2
Rheumatoide Arthritis (*n* = 54.880)	19.042	34,7	2,0	3,4	1	1–2	11.861	21,6	2,1	5,3	1	1–2
Kollagenosen (*n* = 31.322)	9706	31,0	1,9	2,3	1	1–2	6836	21,8	2,2	5,7	1	1–2

*IMID* chronische Entzündungserkrankung(en), *SD* Standardabweichung, *IQR* Interquartilsabstand

Die durchschnittliche Anzahl der Hospitalisierungen pro Patient mit IMID betrug 1,9 (± 2,5) und unterschied sich kaum von der Referenzpopulation mit 2,1 (± 4,7). Innerhalb der einzelnen Krankheitsentitäten lag auch die durchschnittliche Anzahl der Hospitalisierungen am höchsten bei RA-Patienten mit 2,0 (± 3,4) bis hin zur niedrigsten Anzahl bei Spondylitis ankylosans, PsA und Psoriasis mit 1,8 (± 1,5) (Tab. 2).

### Häufigkeit von Facharztkontakten

Unabhängig von der Krankheitsentität und der Facharztdisziplin nahm ein höherer Anteil der Patienten mit IMID ambulante Facharztbehandlungen in Anspruch als in der jeweiligen Referenzpopulation (Tab. 3). Bei 98,0 % der Patienten mit IMID und 90,0 % der Patienten in der Referenzpopulation wurde im Beobachtungszeitraum mindestens ein Besuch beim Allgemeinmediziner dokumentiert; 54,5 % der Patienten mit IMID waren mindestens 1‑mal in ambulanter fachärztlicher Behandlung bei einem Dermatologen, Rheumatologen oder Gastroenterologen im Vergleich zu 25,4 % in der Referenzpopulation.

**Tab. 3:** Inanspruchnahme von ambulanten Facharztdisziplinen durch prävalente Patienten mit IMID und der jeweiligen Referenzpopulation

	Patienten mit IMID						Referenzpopulation					
	Patienten mit ambulanten Kontakten		Anzahl Kontakte				Patienten mit ambulanten Kontakten		Anzahl Kontakte			
	*n*	%	Mittelwert	SD	Median	IQR	*n*	%	Mittelwert	SD	Median	IQR
*Gesamt (n* *=* *188.440)*												
Derma‑, Rheuma‑, Gastroenterologen	102.714	54,5	4,63	6,16	3,00	2–5	47.848	25,4	2,53	2,69	2,00	1–3
Rheumatologen	32.542	17,3	4,15	3,15	3,00	2–5	3315	1,8	2,75	3,19	2,00	1–3
Dermatologen	70.732	37,5	3,88	6,77	2,00	1–4	39.075	20,7	2,38	2,57	2,00	1–3
Gastroenterologen	21.668	11,5	3,10	3,13	2,00	1–4	9819	5,2	1,97	1,74	2,00	1–2
Allgemeinmediziner	184.667	98,0	12,05	8,83	10,00	6–15	169.532	90,0	9,41	8,40	8,00	4–12
*Morbus Crohn (n* *=* *14.455)*												
Derma‑, Rheuma‑, Gastroenterologen	7923	54,8	4,72	4,54	3,00	2–6	3436	23,8	2,36	2,60	3,00	2–6
Rheumatologen	783	5,42	3,40	3,11	2,00	1–4	222	1,5	2,27	2,32	1,00	1–2
Dermatologen	4316	29,9	2,56	3,04	2,00	1–3	2838	19,6	2,22	2,26	1,00	1–3
Gastroenterologen	5036	34,8	4,74	4,13	4,00	2–6	647	4,5	2,02	3,03	2,00	1–2
Allgemeinmediziner	14.101	97,6	11,09	7,97	9,00	6–14	12.549	86,8	7,60	7,67	6,00	3–10
*Colitis ulcerosa (n* *=* *16.790)*												
Derma‑, Rheuma‑, Gastroenterologen	8878	52,9	4,31	4,22	3,00	2–5	4024	24,0	2,41	2,37	2,00	1–3
Rheumatologen	840	5,0	3,36	3,11	2,00	1–4	258	1,5	2,34	2,03	2,00	1–3
Dermatologen	4869	29,0	2,60	3,35	2,00	1–3	3323	19,8	2,27	2,32	1,00	1–3
Gastroenterologen	5496	32,7	4,17	3,64	3,00	2–5	795	4,7	1,96	1,74	2,00	1–2
Allgemeinmediziner	16.426	97,8	11,23	8,34	9,00	6–14	14.764	87,9	8,47	8,07	7,00	3–11
*Psoriasis (n* *=* *73.726)*												
Derma‑, Rheuma‑, Gastroenterologen	42.788	58,0	5,31	8,39	3,00	2–5	18.548	25,2	2,51	2,73	2,00	1–3
Rheumatologen	6710	9,1	3,86	3,13	3,00	2–5	1290	1,8	2,77	3,29	2,00	1–3
Dermatologen	37.641	51,1	5,05	8,72	3,00	2–4	15.059	20,4	2,36	2,66	2,00	1–3
Gastroenterologen	5306	7,2	2,20	2,23	2,00	1–2	3885	5,3	1,95	1,44	2,00	1–2
Allgemeinmediziner	71.990	97,7	11,11	8,62	9,00	6–14	65.885	89,4	9,08	8,33	7,00	4–12
*Psoriasisarthritis (n* *=* *11.493)*												
Derma‑, Rheuma‑, Gastroenterologen	8165	71,0	5,73	7,10	4,00	2–7	2947	25,6	2,51	3,20	2,00	1–3
Rheumatologen	4933	42,9	4,32	3,11	4,00	2–5	192	1,7	2,60	2,61	2,00	1–3
Dermatologen	5085	44,2	4,48	8,12	2,00	1–4	2394	20,8	2,35	3,31	1,00	1–3
Gastroenterologen	1112	9,7	2,63	2,50	2,00	1–3	659	5,7	1,93	1,38	2,00	1–2
Allgemeinmediziner	11.352	98,8	12,37	8,61	11,0	7–16	10.239	89,1	8,47	6,89	7,00	4–11
*Spondylitis ankylosans (n* *=* *11.818)*												
Derma‑, Rheuma‑, Gastroenterologen	6342	53,7	4,13	3,95	3,00	2–5	2956	25,0	2,51	2,80	2,00	1–3
Rheumatologen	3562	30,1	4,08	2,99	3,00	2–5	200	1,7	3,01	3,94	2,00	1–3
Dermatologen	3468	29,4	2,65	3,60	2,00	1–3	2446	20,7	2,31	2,39	1,00	1–3
Gastroenterologen	1102	9,3	2,35	1,98	2,00	1–3	579	4,9	2,05	2,88	2,00	1–2
Allgemeinmediziner	11.665	98,7	11,70	8,56	10,00	6–15	10.559	89,4	8,71	7,80	7,00	4–11
*Rheumatoide Arthritis (n* *=* *54.880)*												
Derma‑, Rheuma‑, Gastroenterologen	31.982	58,3	4,62	4,11	4,00	2–6	14.453	26,3	2,63	2,70	2,00	1–3
Rheumatologen	21.428	39,0	4,47	3,17	4,00	2–6	1053	1,9	2,89	3,32	2,00	1–3
Dermatologen	15.579	28,4	2,75	3,61	2,00	1–3	11.721	21,4	2,46	2,61	2,00	1–3
Gastroenterologen	4485	8,2	2,18	2,24	2,00	1–2	3117	5,7	1,96	1,36	2,00	1–2
Allgemeinmediziner	54.357	99,0	14,04	9,40	12,00	8–18	50.497	92,0	10,30	8,61	8,00	5–13
*Kollagenosen (n* *=* *31.322)*												
Derma‑, Rheuma‑, Gastroenterologen	15.484	49,4	3,78	4,03	3,00	1–5	8248	26,3	2,56	2,69	2,00	1–3
Rheumatologen	5208	16,6	3,96	3,14	3,00	2–5	560	1,8	2,75	3,31	2,00	1–3
Dermatologen	11.008	35,1	2,84	3,68	2,00	1–3	6808	21,7	2,42	2,58	2,00	1–3
Gastroenterologen	3011	9,6	2,26	2,11	2,00	1–3	1592	5,1	1,98	1,69	2,00	1–2
Allgemeinmediziner	30.578	97,6	12,88	8,83	11,00	7–16	28.466	90,9	10,13	8,37	8,00	5–13

*IMID* chronische Entzündungserkrankung(en), *SD* Standardabweichung, *IQR* Interquartilsabstand

Patienten mit PsA, Spondylitis ankylosans und RA waren ≥ 1-mal im Referenzjahr in ambulanter Behandlung bei Rheumatologen (42,9 %, 30,1 % bzw. 39,0 %), Dermatologen (44,2 %, 29,4 % bzw. 28,4 %) und Gastroenterologen (9,7 %, 9,3 % bzw. 8,2 %). Ein ähnlicher Anteil an Gesamtfacharztkontakten war bei Patienten mit Morbus Crohn und Colitis ulcerosa zu verzeichnen: Gastroenterologe (34,8 und 32,7 %), Dermatologen (29,9 und 29,0 %) und Rheumatologen (5,4 und 5,0 %). Bei Patienten mit Kollagenosen und Psoriasis war jeweils die Dermatologie die von den Patienten am häufigsten aufgesuchte ambulante Fachdisziplin (35,1 und 51,1 %), gefolgt von Rheumatologie (16,6 und 9,1 %) und Gastroenterologie (9,6 und 7,2 %).

Die durchschnittliche Anzahl der beobachteten ambulanten Arztkontakte war bei Patienten mit IMID in allen Krankheitsentitäten höher als in der jeweiligen Vergleichspopulation (Tab. 3).

## Diskussion

Bisherige Untersuchungen zur Epidemiologie und Versorgungssituation konzentrierten sich zumeist auf einzelne Entzündungserkrankungen [2, 20–22, 28, 30]. Aufgrund gemeinsamer pathophysiologischer Mechanismen können jedoch mehrere Organe von Entzündungsprozessen betroffen sein [5]. Eine multiple Organbeteiligung, z. B. bei Überlappung von Erkrankungen oder gleichzeitigem Vorliegen mehrerer IMID, kann das diagnostische und therapeutische Versorgungsmanagement der betroffenen Patienten komplexer gestalten. In dieser Studie wurden daher Prävalenzen, demografische Aspekte, Kombinationen von IMID und die Inanspruchnahme ambulanter/stationärer Versorgungsleistungen analysiert, um Hinweise auf den Versorgungsbedarf zu erhalten.

Die in dieser Studie ermittelten Prävalenzen sind im Wesentlichen mit den in der Literatur berichteten vergleichbar. Die in dieser Studie ermittelte Prävalenz für die PsA von 0,29 % liegt im unteren Bereich der für Deutschland publizierten Prävalenzen von 0,2–1,4 % [10], jedoch vollständig im Bereich der in Metaanalysen weltweit berichteten Prävalenzen von 0,1–1 % [24]. Die von uns berechnete Prävalenz für die RA von 1,85 % liegt zwar leicht über den in Deutschland berichteten Prävalenzen von 0,6–1,4 %, allerdings wurde eine vergleichbare Prävalenz von 1,85 % in der NAKO-Gesundheitsstudie berichtet [2]. Unsere ermittelte Prävalenz für Spondylitis ankylosans von 0,3 % liegt dagegen etwas unterhalb der für Deutschland geschätzten Prävalenz von ~0,5 % [10], jedoch innerhalb der weltweit publizierten Prävalenzen von 0,1–1,4 % [15]. Die von uns bestimmten Prävalenzen von 0,36 % für Morbus Crohn und 0,42 % für Colitis ulcerosa stimmen mit den bisher publizierten, methodisch ähnlich ermittelten, Prävalenzen von jeweils 0,32 % bzw. 0,41 % überein [21]. Die Prävalenz für Psoriasis liegt mit 1,9 % etwas unter den Prävalenzen deutscher Daten von 2,0–2,8 % [20].

Unterschiede der hier ermittelten Prävalenzen im Vergleich zu publizierten Daten können v. a. durch unterschiedliche Methodik (z. B. Punkt-, 1‑Jahres- oder Lebenszeitprävalenz; ggf. zusätzliche Kriterien wie spezifische Medikation [3]) und verschiedene Datengrundlagen (Beobachtungsstudien, Register, regionale Erhebungen vs. Krankenkassendaten) erklärt werden.

Insgesamt müssen für den Teil der Prävalenzen wie auch für die folgenden Abschnitte die Limitationen von Abrechnungsdaten klar formuliert werden, insbesondere dass es sich nicht um klinisch validierte Diagnosen handelt. Abrechnungsdiagnosen können fehlerhaft kodiert, bei Diagnoseänderung nicht korrigiert oder unkritisch übernommen worden sein. Sie sind aber auch für epidemiologische Fragestellungen überaus wertvoll, da sie den Vorteil haben, bevölkerungsbezogene Analysen zu ermöglichen und nahezu alle Personen, weitgehend unabhängig von Alter, Geschlecht, sozioökonomischem Status und fachärztlichen Kontakten, einbeziehen [2].

Im Vergleich zur Referenzpopulation wiesen Patienten mit IMID einen höheren Anteil an Hospitalisierungen sowie Inanspruchnahmen von allgemeinmedizinischen Vorstellungen auf, was auf eine höhere Morbidität in der Studienpopulation hindeutet [9]. Dies steht im Einklang mit epidemiologischen Studien, die bei Patienten mit IMID im Vergleich zur Allgemeinbevölkerung eine erhöhte Komorbidität wie kardiovaskuläre Erkrankungen, Osteoporose oder Depressionen gezeigt haben [25, 31]. Es besteht jedoch die Möglichkeit, dass die Anzahl der allgemeinmedizinischen Vorstellungen in unserer Studie etwas überschätzt wird, da ein gewisser Anteil der Patienten (insbesondere bei Beginn oder Umstellung einer Basistherapie) Laborkontrollen beim Allgemeinmediziner durchführen lässt; zudem führt das Fehlen einer IMID-Diagnose in der Referenzpopulation per se zu weniger Arztkontakten, insbesondere mit Fachärzten. Verglichen mit den entsprechenden Referenzpopulationen wiesen Patienten mit IMID zudem mehr Kontakte zu den in die Analyse einbezogenen ambulanten Fachdisziplinen (Dermatologie, Rheumatologie und Gastroenterologie) auf. Dies war aufgrund der notwendigen regelmäßigen Vorstellung beim Facharzt der primären IMID erwartbar. Interessant war jedoch, dass bei den einzelnen Entitäten häufig andere bzw. mehrere Fachdisziplinen in Anspruch genommen wurden. Bei CED erfolgten zu einem beträchtlichen Anteil auch Vorstellungen bei weiteren Disziplinen, z. B. bei 29 % in der Dermatologie und bei ~5 % in der Rheumatologie. Die Rate an Dermatologievorstellungen bei Patienten mit CED ist beispielsweise durchaus plausibel, da nicht nur extraintestinale Manifestationen (Pyoderma gangraenosum, Erythema nodosum), weitere IMIDs (Psoriasis, Psoriasisarthritis, Acne inversa, Cheilitis granulomatosa), sondern durch die notwendigen höheren Medikamentendosen mehr Hautinfektionen (H. zoster, Virusexantheme, Condylomata accuminata, Impetigo) oder Medikamentennebenwirkungen (Steroidakne, Neurodermitis, Psoriasis) auftreten können [19]. Bei den entzündlich rheumatischen Erkrankungen und Psoriasis ergab sich ein ähnliches Bild mit vermehrten Arztbesuchen der jeweils anderen Fachrichtungen. Selbstverständlich kann die Inanspruchnahme der jeweils anderen Fachdisziplinen potenziell auch andere Ursachen haben, denen nicht notwendigerweise eine entzündliche Organmanifestation zugrunde liegen muss. Des Weiteren kann nicht ausgeschlossen werden, dass Codierungen falsch gesetzt oder Diagnosen verkannt werden. Es bleibt aber die Tatsache bestehen, dass Patienten physisch bei einem Facharzt der jeweils anderen Fachdisziplin vorstellig wurden und somit mehr Versorgungsstruktur in Anspruch genommen haben als die Vergleichspopulation. Gerade in dieser Konstellation scheint eine enge Vernetzung der Fachrichtungen aus Patienten- und Arztsicht sowie aus Gründen der Kosteneffektivität (z. B. Vermeidung von Mehrfachuntersuchungen) erstrebenswert. Somit deuten die Ergebnisse zumindest teilweise auf einen multidisziplinären Versorgungsbedarf hin.

Bei der Gesamtbetrachtung der Mehrfachdiagnosen fiel der erwartungsgemäß hohe Anteil der Patienten mit PsA auf, die an einer weiteren IMID litten, was aber im Wesentlichen auf den hohen Anteil an Psoriasis zurückzuführen war, die in der Regel mit der PsA-Diagnose als Haut- und/oder Nagelmanifestation vorhanden ist. Da bei 85–90 % der Patienten mit PsA eine Psoriasis vorausgeht bzw. gleichzeitig auftritt [18], war eine hohe Kombinationsrate dieser beiden Entitäten zu erwarten. In selteneren Fällen kann die Psoriasis auch erst nach dem Beginn der Arthritis auftreten oder die Psoriasis sehr mild ausgeprägt sein bzw. nur die Nägel betreffen, was die Abgrenzung z. B. zur seronegativen RA erschweren kann [24]. Bei über einem Viertel der Patienten mit PsA wurde zusätzlich eine RA-Diagnose dokumentiert. Da die Psoriasis jedoch eher selten mit einer rheumatoider Arthritis assoziiert ist [24], handelt es sich hierbei am ehesten um Fehldiagnosen, verkannte PsA-Diagnosen oder mangelnde Kodierqualität. Auch bei Morbus Crohn und Colitis ulcerosa wurde eine beachtliche Überschneidung beobachtet. Hier ist das gemeinsame Auftreten der Diagnosen am ehesten auf die diagnostischen Unsicherheiten in der Einordnung und Unterscheidung des Krankheitsbildes bei häufig ähnlichen Symptomen zurückzuführen.

Diese Studie weist Limitationen, aber auch Stärken auf. Aufgrund des Querschnittdesigns waren Analysen zu Krankheitsverläufen und kausalen Zusammenhängen, wie z. B. der Einfluss des Schweregrades einer primären IMID auf die Entwicklungsrate weiterer IMID, nicht möglich. Die zugrunde liegende Datenbasis enthielt keine Informationen zum Schweregrad der Erkrankung oder der Frage, ob Facharztkontakte tatsächlich zur Behandlung der betrachteten IMID erfolgten. Eine Stärke der vorliegenden Arbeit ist die repräsentative Datenbasis. Real-World-Daten haben allgemein den Vorteil, das Versorgungsgeschehen authentisch abbilden und in der Regel nicht durch systematische Designschwachpunkte verzerrt sind, die bei randomisiert kontrollierten Studien (RCT) durch restriktive Ein- und Ausschlusskriterien vorkommen können [1, 4, 13]. Die InGef-Datenbank enthält Abrechnungsdaten verschiedener gesetzlicher Krankenkassen, hauptsächlich Betriebskrankenkassen und Innungskrankenkassen. Es ist bekannt, dass sowohl die soziodemografischen Informationen als auch die Informationen zur Morbidität zwischen den verschiedenen Arten von GKV-Unternehmen variieren. Wenn diese Unterschiede zwischen den Krankenkassen mit den in dieser Studie untersuchten Indikatoren korrelieren und diese Faktoren in der Analysestichprobe systematisch verzerrt würden (z. B. Überrepräsentation von Personen mit höherem sozioökonomischem Status), könnte dies zu verzerrten Schätzungen führen. Rezente Publikationen weisen jedoch auf eine hohe externe Validität der InGef-Datenbank in Bezug auf Morbidität, Mortalität und Medikamenteneinnahme hin [6].

Die vorliegende Studie bietet trotz der genannten Limitationen bezüglich der Methodik und daraus resultierender Notwendigkeit der vorsichtigen Interpretation eine disziplinübergreifende Betrachtung von Epidemiologie und Versorgungssituation von IMID in Deutschland unter Verwendung von aktuellen Real-World-Daten.

Die Studienergebnisse deuten auf eine hohe Morbidität und einen erhöhten multidisziplinären Versorgungsbedarf der betroffenen Patienten hin. Um irreversible Organschäden bei IMID zu vermeiden, sind eine frühzeitige Diagnosestellung sowie eine optimale Therapiesteuerung mit bedarfsgerechter interdisziplinärer Koordination von zentraler Bedeutung. Dies könnte zukünftig verstärkt in interdisziplinären Entzündungsboards bzw. Entzündungszentren als ergänzende Versorgungsstruktur erfolgen, deren Sinnhaftigkeit teilweise auch durch unsere Daten gestützt wird. Um weitere Erkenntnisse über den Krankheits- und Therapieverlauf bei Patienten mit IMID für das deutsche Versorgungssetting zu gewinnen, sind jedoch weitere Studien auf der Basis von Längsschnittanalysen erforderlich.

## Supplementary Information

ESM 1: Tab. S1: Operationalisierung der IMID/Tab. S2: 12-Monats-Prävalenz des Auftretens von mindestens einer IMID, stratifiziert nach Alter und Geschlecht/Tab. S3:12-Monats-Prävalenz der Psoriasis, stratifiziert nach Alter und Geschlecht/Tab. S4: 12-Monats-Prävalenz der rheumatoiden Arthritis, stratifiziert nach Alter und Geschlecht/Tab. S5: 12-Monats-Prävalenz der Colitis ulcerosa, stratifiziert nach Alter und Geschlecht/Tab. S6: 12-Monats-Prävalenz des Morbus Crohn, stratifiziert nach Alter und Geschlecht/Tab. S7: 12-Monats-Prävalenz der Spondylitis ankylosans, stratifiziert nach Alter und Geschlecht/Tab. S8: 12-Monats-Prävalenz der Psoriasisarthritis, stratifiziert nach Alter und Geschlecht/Tab. S9: 12-Monats-Prävalenz der Kollagenosen, stratifiziert nach Alter und Geschlecht

ESM 2: STROBE Statement—checklist of items that should be included in reports of observational studies
