# Supplementary material for: Epidemiological data and medical care situation of patients with chronic inflammatory diseases in Germany: Real-world evidence on prevalence, disease combinations, care
Source: Z Rheumatol. 2023 Dec 9;83(7):578–86. [Article in German] doi: 10.1007/s00393-023-01459-7 (PMC11442525; doi:10.1007/s00393-023-01459-7)
Supplement: Supplementary file 1 — ESM 1: Tab. S1: Operationalisierung der IMID/Tab. S2: 12-Monats-Prävalenz des Auftretens von mindestens einer IMID, stratifiziert nach Alter und Geschlecht/Tab. S3:12-Monats-Prävalenz der Psoriasis, stratifiziert nach Alter und Geschlecht/Tab. S4: 12-Monats-Prävalenz der rheumatoiden Arthritis, stratifiziert nach Alter und Geschlecht/Tab. S5: 12-Monats-Prävalenz der Colitis ulcerosa, stratifiziert nach Alter und Geschlecht/Tab. S6: 12-Monats-Prävalenz des Morbus Crohn, stratifiziert nach Alter und Geschlecht/Tab. S7: 12-Monats-Prävalenz der Spondylitis ankylosans, stratifiziert nach Alter und Geschlecht/Tab. S8: 12-Monats-Prävalenz der Psoriasisarthritis, stratifiziert nach Alter und Geschlecht/Tab. S9: 12-Monats-Prävalenz der Kollagenosen, stratifiziert nach Alter und Geschlecht [file 393_2023_1459_MOESM1_ESM.docx]

**Ergänzende Tabellen**

Tabelle S1: Operationalisierung der IMID.

| IMID | Operationalisierung (ICD-10-GM-Kode) |
| --- | --- |
| Psoriasis | L40 – exklusive L40.5 |
| Psoriasis Arthritis | L40.5, M09.0, M07.0, M07.1, M07.2, M07.3, M07.4, M07.5, M07.6 |
| Rheumatoide Arthritis | M05, M06 |
| Spondylitis ankylosans | M45, M46 [nicht als Aufgreifkriterium] |
| Colitis ulcerosa | K51 |
| Morbus Crohn | K50 |
| Kollagenosen |  |
| Systemischer Lupus Erythematodes | M32 |
| Systemische Sklerose | M34 |
| Dermatomyositis / Polymyositis | M33 |
| Andere Erkrankungen mit systemischer Beteiligung des Bindegewebes | M35 – exklusive M35.3 |

***IMID*** – Chronische Entzündungserkrankung(en)

Tabelle S2: 12-Monats-Prävalenz des Auftretens von mindestens einer IMID, stratifiziert nach Alter und Geschlecht.

|  | Frauen (n=108.454) | | | | Männer (n=79.986) | | | | Gesamt (n=188.440) | | | |
| --- | --- | --- | --- | --- | --- | --- | --- | --- | --- | --- | --- | --- |
|  |  | | 95%-CI | |  | | 95%-CI | |  |  | 95%-CI | |
|  | n | Pro 100.000 | Min | Max | n | Pro 100.000 | Min | Max | n | Pro 100.000 | Min | Max |
| Alter |  |  |  |  |  |  |  |  |  |  |  |  |
| 1-10 Jahre | 302 | 172 | 153,5 | 192,9 | 267 | 144 | 127,6 | 162,8 | 569 | 158 | 145,3 | 171,5 |
| 11-20 Jahre | 1.453 | 818 | 776,3 | 860,6 | 1.137 | 597 | 562,7 | 632,4 | 2.590 | 703 | 676,6 | 730,9 |
| 21-30 Jahre | 4.509 | 2.115 | 2.054,0 | 2.176,6 | 3.477 | 1.512 | 1.462,2 | 1.562,4 | 7.986 | 1.802 | 1.762,8 | 1.841,3 |
| 31-40 Jahre | 7.783 | 3.224 | 3.153,8 | 3.295,1 | 6.325 | 2.537 | 2.475,5 | 2.599,3 | 14.108 | 2.875 | 2.828,2 | 2.922,0 |
| 41-50 Jahre | 11.717 | 4.529 | 4.448,8 | 4.609,4 | 9.877 | 3.768 | 3.695,4 | 3.841,6 | 21.594 | 4.146 | 4.091,8 | 4.200,3 |
| 51-60 Jahre | 21.962 | 6.703 | 6.618,0 | 6.789,6 | 17.820 | 5.385 | 5.307,9 | 5.462,0 | 39.782 | 6.041 | 5.983,3 | 6.098,5 |
| 61-70 Jahre | 24.964 | 9.190 | 9.081,5 | 9.299,1 | 17.821 | 7.307 | 7.204,5 | 7.411,5 | 42.785 | 8.299 | 8.224,2 | 8.375,0 |
| 71-80 Jahre | 22.062 | 10.579 | 10.447,5 | 10.712,0 | 15.253 | 8.452 | 8.324,5 | 8.581,7 | 37.315 | 9.593 | 9.500,2 | 9.685,5 |
| 81-90 Jahre | 12.142 | 9.883 | 9.716,4 | 10.050,9 | 7.431 | 8.557 | 8.371,8 | 8.745,0 | 19.573 | 9.334 | 9.209,5 | 9.459,0 |
| ≥ 91 Jahre | 1.560 | 6.737 | 6.417,6 | 7.067,7 | 578 | 6.066 | 5.594,7 | 6.563,8 | 2.138 | 6.541 | 6.275,7 | 6.814,9 |
| Gesamt | 108.454 | 5.369 | 5.337,6 | 5.399,8 | 79.986 | 4.063 | 4.035,7 | 4.090,9 | 188.440 | 4.724 | 4.703,6 | 4.745,2 |

***IMID*** – Chronische Entzündungserkrankung(en), ***CI*** – Konfidenzintervall

Tabelle S3: 12-Monats-Prävalenz der Psoriasis, stratifiziert nach Alter und Geschlecht.

|  | Frauen (n=108.454) | | | | Männer (n=79.986) | | | | Gesamt (n=188.440) | | | |
| --- | --- | --- | --- | --- | --- | --- | --- | --- | --- | --- | --- | --- |
|  |  |  | 95%-CI | |  |  | 95%-CI | |  |  | 95%-CI | |
|  | n | Pro 100.000 | Min | Max | n | Pro 100.000 | Min | Max | n | Pro 100.000 | Min | Max |
| Alter |  |  |  |  |  |  |  |  |  |  |  |  |
| 1-10 Jahre | 117 | 67 | 55,2 | 80,0 | 84 | 45 | 36,2 | 56,2 | 201 | 56 | 48,4 | 64,1 |
| 11-20 Jahre | 511 | 288 | 263,2 | 313,5 | 431 | 226 | 205,4 | 248,6 | 942 | 256 | 239,8 | 272,7 |
| 21-30 Jahre | 1.536 | 720 | 684,9 | 757,2 | 1.432 | 623 | 590,9 | 655,6 | 2,968 | 670 | 645,8 | 694,1 |
| 31-40 Jahre | 2.859 | 1.184 | 1.141,5 | 1.228,2 | 2.999 | 1.203 | 1.160,4 | 1.246,4 | 5.858 | 1.194 | 1.163,5 | 1.224,5 |
| 41-50 Jahre | 4.291 | 1.658 | 1.609,6 | 1.708,4 | 5.036 | 1.921 | 1.869,0 | 1.974,5 | 9.327 | 1.791 | 1.754,8 | 1.827,1 |
| 51-60 Jahre | 8.029 | 2.451 | 2.398,0 | 2.504,2 | 9.113 | 2.754 | 2.698,1 | 2.810,0 | 17.142 | 2.603 | 2.564,6 | 2.641,7 |
| 61-70 Jahre | 8.875 | 3.267 | 3.200,6 | 3.334,6 | 8.993 | 3.688 | 3.613,1 | 3.763,1 | 17.868 | 3.466 | 3.416,2 | 3.516,3 |
| 71-80 Jahre | 6.409 | 3.073 | 2.999,6 | 3.148,2 | 6.392 | 3.542 | 3.457,3 | 3.628,4 | 12.801 | 3.291 | 3.234,9 | 3.347,3 |
| 81-90 Jahre | 3.181 | 2.589 | 2.501,0 | 2.679,4 | 2.794 | 3.217 | 3.101,0 | 3.336,9 | 5.975 | 2.849 | 2.778,5 | 2.921,4 |
| ≥ 91 Jahre | 429 | 1.853 | 1.682,9 | 2.034,7 | 215 | 2.256 | 1.967,5 | 2.574,7 | 644 | 1.970 | 1.822,5 | 2.126,9 |
| Gesamt | 36.237 | 1.794 | 1.775,5 | 1.812,2 | 37.489 | 1.904 | 1.885,4 | 1.923,6 | 73.726 | 1.848 | 1.835,2 | 1.861,6 |

***CI*** – Konfidenzintervall

Tabelle S4: 12-Monats-Prävalenz der rheumatoiden Arthritis, stratifiziert nach Alter und Geschlecht.

|  | Frauen (n=108.454) | | | | Männer (n=79.986) | | | | Gesamt (n=188.440) | | | |
| --- | --- | --- | --- | --- | --- | --- | --- | --- | --- | --- | --- | --- |
|  |  | | 95%-CI | |  |  | 95%-CI | |  |  | 95%-CI | |
|  | n | Pro 100.000 | Min | Max | n | Pro 100.000 | Min | Max | n | Pro 100.000 | Min | Max |
| Alter |  |  |  |  |  |  |  |  |  |  |  |  |
| 1-10 Jahre | 30 | 17 | 11,6 | 24,4 | 10 | 5 | 2,6 | 9,9 | 40 | 11 | 7,9 | 15,1 |
| 11-20 Jahre | 141 | 79 | 66,8 | 93,6 | 75 | 39 | 31,0 | 49,3 | 216 | 59 | 51,1 | 67,0 |
| 21-30 Jahre | 663 | 311 | 287,7 | 335,5 | 211 | 92 | 79,8 | 105,0 | 874 | 197 | 184,3 | 210,7 |
| 31-40 Jahre | 1.381 | 572 | 542,3 | 602,9 | 528 | 212 | 194,1 | 230,6 | 1.909 | 389 | 371,8 | 406,8 |
| 41-50 Jahre | 3.030 | 1.171 | 1.130,0 | 1.213,3 | 1.293 | 493 | 466,8 | 520,8 | 4,323 | 830 | 805,5 | 855,0 |
| 51-60 Jahre | 7.271 | 2.219 | 2.169,1 | 2.270,3 | 3.592 | 1.085 | 1.050,4 | 1.121,3 | 10.863 | 1.649 | 1.618,9 | 1.680,5 |
| 61-70 Jahre | 9.699 | 3.570 | 3.501,0 | 3.640,9 | 4.177 | 1.713 | 1.661,7 | 1.765,1 | 13.876 | 2.692 | 2.647,6 | 2.736,2 |
| 71-80 Jahre | 9.535 | 4.572 | 4.483,0 | 4.662,7 | 4.411 | 2.444 | 2.373,6 | 2.516,6 | 13.946 | 3.585 | 3.526,9 | 3.644,0 |
| 81-90 Jahre | 5.592 | 4.551 | 4.435,6 | 4.669,5 | 2.294 | 2.642 | 2.536,0 | 2.750,4 | 7.886 | 3.761 | 3.679,6 | 3.842,9 |
| ≥ 91 Jahre | 731 | 3.157 | 2.935,5 | 3.390,3 | 216 | 2.267 | 1.977,3 | 2.585,9 | 947 | 2.897 | 2.718,3 | 3.085,1 |
| Gesamt | 38.073 | 1.885 | 1.866,0 | 1.903,5 | 16.807 | 854 | 841,0 | 866,7 | 54.880 | 1.376 | 1.364,5 | 1.387,4 |

***CI*** – Konfidenzintervall

Tabelle S5: 12-Monats-Prävalenz der Colitis ulcerosa, stratifiziert nach Alter und Geschlecht.

|  | Frauen (n=108.454) | | | | Männer (n=79.986) | | | | Gesamt (n=188.440) | | | |
| --- | --- | --- | --- | --- | --- | --- | --- | --- | --- | --- | --- | --- |
|  |  |  | 95%-CI | |  |  | 95%-CI | |  |  | 95%-CI | |
|  | n | Pro 100.000 | Min | Max | n | Pro 100.000 | Min | Max | n | Pro 100.000 | Min | Max |
| Alter |  |  |  |  |  |  |  |  |  |  |  |  |
| 1-10 Jahre | 30 | 17 | 11,6 | 24,4 | 30 | 16 | 10,9 | 23,2 | 60 | 17 | 12,7 | 21,4 |
| 11-20 Jahre | 194 | 109 | 94,3 | 125,6 | 187 | 98 | 84,6 | 113,3 | 381 | 103 | 93,3 | 114,4 |
| 21-30 Jahre | 651 | 305 | 282,3 | 329,6 | 665 | 289 | 267,6 | 311,9 | 1.316 | 297 | 281,1 | 313,4 |
| 31-40 Jahre | 1.084 | 449 | 422,7 | 476,5 | 1.067 | 428 | 402,7 | 454,4 | 2.151 | 438 | 420,0 | 457,2 |
| 41-50 Jahre | 1.323 | 511 | 484,2 | 539,6 | 1.335 | 509 | 482,4 | 537,3 | 2.658 | 510 | 491,1 | 530,0 |
| 51-60 Jahre | 1.798 | 549 | 523,8 | 574,7 | 1.960 | 592 | 566,4 | 619,0 | 3.758 | 571 | 552,6 | 589,1 |
| 61-70 Jahre | 1.499 | 552 | 524,3 | 580,4 | 1.543 | 633 | 601,6 | 665,0 | 3.042 | 590 | 569,4 | 611,4 |
| 71-80 Jahre | 1.034 | 496 | 466,1 | 526,9 | 1.164 | 645 | 608,6 | 683,0 | 2.198 | 565 | 541,7 | 589,1 |
| 81-90 Jahre | 628 | 511 | 472,0 | 552,6 | 493 | 568 | 518,8 | 619,9 | 1.121 | 535 | 503,8 | 566,7 |
| ≥ 91 Jahre | 74 | 320 | 251,0 | 401,0 | 31 | 325 | 221,1 | 461,5 | 105 | 321 | 262,8 | 388,8 |
| Gesamt | 8.315 | 412 | 402,8 | 420,5 | 8.475 | 431 | 421,4 | 439,8 | 16.790 | 421 | 414,6 | 427,3 |

***CI*** – Konfidenzintervall

Tabelle S6: 12-Monats-Prävalenz des Morbus Crohn, stratifiziert nach Alter und Geschlecht.

|  | Frauen (n=108.454) | | | | Männer (n=79.986) | | | | Gesamt (n=188.440) | | | |
| --- | --- | --- | --- | --- | --- | --- | --- | --- | --- | --- | --- | --- |
|  |  |  | 95%-CI | |  |  | 95%-CI | |  |  | 95%-CI | |
|  | n | Pro 100.000 | Min | Max | n | Pro 100.000 | Min | Max | n | Pro 100.000 | Min | Max |
| Alter |  |  |  |  |  |  |  |  |  |  |  |  |
| 1-10 Jahre | 28 | 16 | 10,6 | 23,1 | 36 | 19 | 13,6 | 26,9 | 64 | 18 | 13,7 | 22,7 |
| 11-20 Jahre | 241 | 136 | 119,0 | 153,8 | 279 | 146 | 129,8 | 164,7 | 520 | 141 | 129,3 | 153,9 |
| 21-30 Jahre | 930 | 436 | 408,6 | 465,0 | 792 | 344 | 320,8 | 369,1 | 1.722 | 389 | 370,4 | 407,3 |
| 31-40 Jahre | 1.279 | 530 | 501,2 | 559,6 | 1.040 | 417 | 392,2 | 443,2 | 2.319 | 473 | 453,6 | 492,1 |
| 41-50 Jahre | 1.283 | 496 | 469,2 | 523,7 | 1.087 | 415 | 390,4 | 440,0 | 2.370 | 455 | 436,9 | 473,7 |
| 51-60 Jahre | 1.937 | 591 | 565,3 | 618,1 | 1.412 | 427 | 404,7 | 449,5 | 3.349 | 509 | 491,5 | 526,0 |
| 61-70 Jahre | 1.362 | 501 | 475,2 | 528,7 | 941 | 386 | 361,6 | 411,3 | 2.303 | 447 | 428,7 | 465,3 |
| 71-80 Jahre | 719 | 345 | 320,1 | 370,9 | 536 | 297 | 272,4 | 323,2 | 1.255 | 323 | 305,0 | 340,9 |
| 81-90 Jahre | 296 | 241 | 214,3 | 270,0 | 209 | 241 | 209,2 | 275,6 | 505 | 241 | 220,3 | 262,7 |
| ≥ 91 Jahre | 36 | 155 | 108,9 | 215,2 | 12 | 126 | 65,1 | 219,9 | 48 | 147 | 108,3 | 194,7 |
| Gesamt | 8.111 | 402 | 392,8 | 410,3 | 6.344 | 322 | 314,4 | 330,3 | 14.455 | 362 | 356,5 | 368,3 |

***CI*** – Konfidenzintervall

Tabelle S7: 12-Monats-Prävalenz der Spondylitis ankylosans, stratifiziert nach Alter und Geschlecht.

|  | Frauen (n=108.454) | | | | Männer (n=79.986) | | | | Gesamt (n=188.440) | | | |
| --- | --- | --- | --- | --- | --- | --- | --- | --- | --- | --- | --- | --- |
|  |  |  | 95%-CI | |  |  | 95%-CI | |  |  | 95%-CI | |
|  | n | Pro 100.000 | Min | Max | n | Pro 100.000 | Min | Max | n | Pro 100.000 | Min | Max |
| Alter |  |  |  |  |  |  |  |  |  |  |  |  |
| 1-10 Jahre | / | / | / | 2,1 | / | / | / | 2,0 | / | / | / | 1,0 |
| 11-20 Jahre | 24 | 14 | 8,7 | 20,1 | 27 | 14 | 9,3 | 20,6 | 51 | 14 | 10,3 | 18,2 |
| 21-30 Jahre | 213 | 100 | 86,9 | 114,2 | 291 | 127 | 112,4 | 141,9 | 504 | 114 | 104,0 | 124,1 |
| 31-40 Jahre | 493 | 204 | 186,6 | 223,0 | 671 | 269 | 249,2 | 290,3 | 1.164 | 237 | 223,8 | 251,2 |
| 41-50 Jahre | 827 | 320 | 298,2 | 342,1 | 1.047 | 399 | 375,6 | 424,3 | 1.874 | 360 | 343,7 | 376,4 |
| 51-60 Jahre | 1.284 | 392 | 370,8 | 413,9 | 1.690 | 511 | 486,7 | 535,5 | 2.974 | 452 | 435,5 | 468,1 |
| 61-70 Jahre | 1.018 | 375 | 352,1 | 398,4 | 1.471 | 603 | 572,8 | 634,7 | 2.489 | 483 | 464,1 | 502,1 |
| 71-80 Jahre | 631 | 303 | 279,5 | 327,1 | 1.215 | 673 | 636,1 | 712,1 | 1.846 | 475 | 453,2 | 496,6 |
| 81-90 Jahre | 290 | 236 | 209,7 | 264,8 | 543 | 625 | 573,9 | 680,0 | 833 | 397 | 370,8 | 425,1 |
| ≥ 91 Jahre | 50 | 216 | 160,3 | 284,6 | 33 | 346 | 238,5 | 486,0 | 83 | 254 | 202,3 | 314,7 |
| Gesamt | 4.830 | 239 | 232,4 | 245,9 | 6.988 | 355 | 346,7 | 363,4 | 11.818 | 296 | 291,0 | 301,7 |

***CI*** – Konfidenzintervall

Tabelle S8: 12-Monats-Prävalenz der Psoriasis-Arthritis, stratifiziert nach Alter und Geschlecht.

|  | Frauen (n=108.454) | | | | Männer (n=79.986) | | | | Gesamt (n=188.440) | | | |
| --- | --- | --- | --- | --- | --- | --- | --- | --- | --- | --- | --- | --- |
|  |  |  | 95%-CI | |  |  | 95%-CI | |  |  | 95%-CI | |
|  | n | Pro 100.000 | Min | Max | n | Pro 100.000 | Min | Max | n | Pro 100.000 | Min | Max |
| Alter |  |  |  |  |  |  |  |  |  |  |  |  |
| 1-10 Jahre | 7 | 4 | 1,6 | 8,2 | <5 | / | / | / | 10 | 3 | 1,3 | 5,1 |
| 11-20 Jahre | 54 | 30 | 22,8 | 39,6 | 21 | 11 | 6,8 | 16,8 | 75 | 20 | 16,0 | 25,5 |
| 21-30 Jahre | 188 | 88 | 76,0 | 101,7 | 141 | 61 | 51,6 | 72,3 | 329 | 74 | 66,4 | 82,7 |
| 31-40 Jahre | 443 | 184 | 166,8 | 201,4 | 374 | 150 | 135,2 | 166,0 | 817 | 166 | 155,3 | 178,3 |
| 41-50 Jahre | 858 | 332 | 309,8 | 354,5 | 835 | 319 | 297,3 | 340,9 | 1.693 | 325 | 309,8 | 340,9 |
| 51-60 Jahre | 1.787 | 545 | 520,5 | 571,3 | 1.516 | 458 | 435,4 | 481,7 | 3.303 | 502 | 484,6 | 518,9 |
| 61-70 Jahre | 1.768 | 651 | 620,9 | 681,8 | 1.269 | 520 | 492,2 | 549,7 | 3.037 | 589 | 568,4 | 610,4 |
| 71-80 Jahre | 985 | 472 | 443,4 | 502,7 | 674 | 373 | 345,9 | 402,7 | 1.659 | 426 | 406,2 | 447,5 |
| 81-90 Jahre | 326 | 265 | 237,3 | 295,7 | 204 | 235 | 203,8 | 269,4 | 530 | 253 | 231,7 | 275,2 |
| ≥ 91 Jahre | 30 | 130 | 87,4 | 184,9 | 10 | 105 | 50,3 | 192,9 | 40 | 122 | 87,4 | 166,6 |
| Gesamt | 6.446 | 319 | 311,4 | 327,0 | 5.047 | 256 | 249,4 | 263,5 | 11.493 | 288 | 282,9 | 293,4 |

***CI*** – Konfidenzintervall

Tabelle S9: 12-Monats-Prävalenz der Kollagenosen, stratifiziert nach Alter und Geschlecht.

|  | Frauen (n=108.454) | | | | Männer (n=79.986) | | | | Gesamt (n=188.440) | | | |
| --- | --- | --- | --- | --- | --- | --- | --- | --- | --- | --- | --- | --- |
|  |  |  | 95%-CI | |  |  | 95%-CI | |  |  | 95%-CI | |
|  | n | Pro 100.000 | Min | Max | n | Pro 100.000 | Min | Max | n | Pro 100.000 | Min | Max |
| Alter |  |  |  |  |  |  |  |  |  |  |  |  |
| 1-10 Jahre | 107 | 61 | 50,0 | 73,8 | 115 | 62 | 51,3 | 74,6 | 222 | 62 | 53,8 | 70,3 |
| 11-20 Jahre | 385 | 217 | 195,6 | 239,4 | 178 | 93 | 80,2 | 108,2 | 563 | 153 | 140,5 | 166,0 |
| 21-30 Jahre | 794 | 372 | 347,0 | 399,1 | 237 | 103 | 90,3 | 117,0 | 1.031 | 233 | 218,6 | 247,2 |
| 31-40 Jahre | 1.279 | 530 | 501,2 | 559,6 | 375 | 150 | 135,6 | 166,4 | 1.654 | 337 | 321,0 | 353,7 |
| 41-50 Jahre | 2.026 | 783 | 749,4 | 817,8 | 661 | 252 | 233,3 | 272,1 | 2.687 | 516 | 496,6 | 535,7 |
| 51-60 Jahre | 3.780 | 1.154 | 1.117,5 | 1.190,9 | 1.336 | 404 | 382,4 | 425,9 | 5.116 | 777 | 755,8 | 798,3 |
| 61-70 Jahre | 4.886 | 1.799 | 1.749,0 | 1.849,3 | 1.923 | 789 | 753,8 | 824,4 | 6.809 | 1.321 | 1.289,8 | 1.352,3 |
| 71-80 Jahre | 5.609 | 2.690 | 2.620,6 | 2.760,0 | 2.688 | 1.490 | 1.434,2 | 1.546,5 | 8.297 | 2.133 | 2.087,7 | 2.178,8 |
| 81-90 Jahre | 2.994 | 2.437 | 2.351,4 | 2.524,7 | 1.530 | 1.762 | 1.675,4 | 1.851,5 | 4.524 | 2.157 | 2.095,6 | 2.220,4 |
| ≥ 91 Jahre | 323 | 1.395 | 1.247,9 | 1.554,4 | 96 | 1.007 | 816,8 | 1.228,9 | 419 | 1.282 | 1.162,8 | 1.409,9 |
| Gesamt | 22.183 | 1.098 | 1.083,8 | 1.112,6 | 9.139 | 464 | 454,8 | 473,8 | 3.,322 | 785 | 776,6 | 794,0 |

***CI*** – Konfidenzintervall
