# Supplementary material for: Epidemiological data and medical care situation of patients with chronic inflammatory diseases in Germany: Real-world evidence on prevalence, disease combinations, care
Source: Z Rheumatol. 2023 Dec 9;83(7):578–86. [Article in German] doi: 10.1007/s00393-023-01459-7 (PMC11442525; doi:10.1007/s00393-023-01459-7)
Supplement: Supplementary file 2 — ESM 2: STROBE Statement—checklist of items that should be included in reports of observational studies [file 393_2023_1459_MOESM2_ESM.docx]

STROBE Statement—checklist of items that should be included in reports of observational studies

|  | Item No. | Recommendation | Page  No. | Relevant text from manuscript |
| --- | --- | --- | --- | --- |
| **Title and abstract** | 1 | (*a*) Indicate the study’s design with a commonly used term in the title or the abstract | 3 | Retrospektive Querschnittsanalyse |
|  |  | (*b*) Provide in the abstract an informative and balanced summary of what was done and what was found | 3 | Basierend auf Abrechnungsdaten der (deutschen) gesetzlichen Krankenversicherung, wurde eine retrospektive Querschnittsanalyse für das Kalenderjahr 2018 durchgeführt.  Insgesamt wurden 188.440 Patienten mit IMID (4,7%) identifiziert. […] Patienten mit IMID wurden verglichen zur Referenzpopulation häufiger hospitalisiert und wiesen häufigere Inanspruchnahmen der betrachteten ambulanten Fachdisziplinen auf. |
| Introduction | | | |  |
| Background/rationale | 2 | Explain the scientific background and rationale for the investigation being reported | 4 | Laut dem aktuellen Stand der Forschung verfügen IMID über gemeinsame pathogene Mechanismen. […] Disziplinübergreifende Ansätze zur Untersuchung von Epidemiologie und Versorgungssituation sind bis dato allerdings für dieses breite Krankheitsspektrum in der Forschungsliteratur kaum verfügbar und eine gesamtheitliche Betrachtung von IMID ist selten. |
| Objectives | 3 | State specific objectives, including any prespecified hypotheses | 5 | Ziel der vorliegenden Studie war es, den Bedarf an einer interdisziplinären Versorgung von IMID in Deutschland zu beleuchten. |
| Methods | | | |  |
| Study design | 4 | Present key elements of study design early in the paper | 6 | Zur Analyse von Krankheitslast und Versorgungssituation wurde eine retrospektive Querschnittstudie mit dem Kalenderjahr 2018 als Beobachtungszeitraum durchgeführt. |
| Setting | 5 | Describe the setting, locations, and relevant dates, including periods of recruitment, exposure, follow-up, and data collection | 6 | Die vorliegende Studie basierte auf der Datenbank des Instituts für angewandte Gesundheitsforschung Berlin (InGef). |
| Participants | 6 | (*a*) *~~Cohort study~~*~~—Give the eligibility criteria, and the sources and methods of selection of participants. Describe methods of follow-up~~  *~~Case-control study~~*~~—Give the eligibility criteria, and the sources and methods of case ascertainment and control selection. Give the rationale for the choice of cases and controls~~  *Cross-sectional study*—Give the eligibility criteria, and the sources and methods of selection of participants | 6 | Die Studienpopulation umfasste prävalente Patienten mit mindestens zwei gesicherten ambulanten IMID-Diagnosen in zwei Quartalen (M2Q) beziehungsweise mit mindestens einer stationären Haupt- oder Nebendiagnose im Studienjahr. Versicherte mussten im Beobachtungszeitraum durchgängig beziehungsweise durchgängig bis zum Tod versichert sein. |
|  |  | ~~(~~*~~b~~*~~)~~ *~~Cohort study~~*~~—For matched studies, give matching criteria and number of exposed and unexposed~~  *~~Case-control study~~*~~—For matched studies, give matching criteria and the number of controls per case~~ |  |  |
| Variables | 7 | Clearly define all outcomes, exposures, predictors, potential confounders, and effect modifiers. Give diagnostic criteria, if applicable | 6 | Insgesamt wurden sieben IMID-Entitäten berücksichtigt (ICD-10-GM): Psoriasis (L40. - ohne L40.5), Psoriasis-Arthritis (L40.5 oder M07.-), rheumatoide Arthritis (M05.- oder M06.-), Spondylitis ankylosans (M45.- und M46.-), Colitis ulcerosa (K51.-), Morbus Crohn (K50.-) und Kollagenosen. Die Gruppe der Kollagenosen umfasste Systemischer Lupus erythematodes (M32.-), Systemische Sklerose (M34.-), Dermatomyositis / Polymyositis (M33.-) und andere Erkrankungen mit systemischer Beteiligung des Bindegewebes (zum Beispiel M35.- ohne M35.3), die jedoch nicht als Subgruppen im Rahmen der Studien analysiert wurden. |
| Data sources/ measurement | 8* | For each variable of interest, give sources of data and details of methods of assessment (measurement). Describe comparability of assessment methods if there is more than one group | 6 | Enthalten waren Daten zu Arzneimittelverschreibungen sowie zu ambulanten und stationären Diagnose- und Leistungsdaten. Diagnosen wurden gemäß der deutschen Version der Internationalen statistischen Klassifikation der Krankheiten und verwandter Gesundheitsprobleme in der 10. Revision (ICD-10-GM) dokumentiert. |
| Bias | 9 | Describe any efforts to address potential sources of bias | - | Die Ergebnisse dieser Studie zeigen eine generelle Kassendatenanalyse. Es besteht kein Grund zur Voreingenommenheit. |
| Study size | 10 | Explain how the study size was arrived at | 6 | Die Datenbank umfasst aggregierte, anonymisierte GKV-Längsschnittdaten von circa 60 Krankenkassen und bundesweit etwa acht Millionen Versicherten. |

Continued on next page

| Quantitative variables | 11 | Explain how quantitative variables were handled in the analyses. If applicable, describe which groupings were chosen and why | 7 | Innerhalb der Studienpopulation wurden Kombinationen (Dokumentation mehrerer IMID pro Kalenderjahr (M2Q)) ermittelt, wobei die Dokumentation einer weiteren IMID-Diagnose nach den zuvor beschriebenen Kriterien innerhalb des Studienzeitraums ausreichend war, um als Kombination identifiziert zu werden. |
| --- | --- | --- | --- | --- |
| Statistical methods | 12 | (*a*) Describe all statistical methods, including those used to control for confounding | 7 | Für die Studienpopulation sowie die einzelnen IMID-Entitäten wurden die absolute und relative Häufigkeit der Inanspruchnahme von Hospitalisierungen und ambulaten Versorgungsleistungen erfasst. Ebenso wurde die mittlere Anzahl der Arztkontakte pro Patient ermittelt und jeweils mit einer korrespondierenden alters- und geschlechtsadjustierten Referenzpopulation ohne IMID verglichen. Es wurden voll-, teil- und vorstationäre Aufenthalte und ambulante Arztkontakte in den Fachdisziplinen Allgemeinmedizin, Dermatologie, Rheumatologie und Gastroenterologie berücksichtigt |
|  |  | (*b*) Describe any methods used to examine subgroups and interactions | 7 | Die definierten Krankheitsentitäten wurden sowohl einzeln als auch als aggregierte Gesamtgruppe analysiert. Zur Bestimmung der Krankheitslast, wurden 12-Monats-Prävalenzen für IMID ermittelt. |
|  |  | (*c*) Explain how missing data were addressed | - | Da diese Querschnittstudie auf einer Datenbank basiert, gibt es keine „fehlenden Daten“, die berücksichtigt werden müssten. |
|  |  | (*d*) *~~Cohort study~~*~~—If applicable, explain how loss to follow-up was addressed~~  *~~Case-control study~~*~~—If applicable, explain how matching of cases and controls was addressed~~  *Cross-sectional study*—If applicable, describe analytical methods taking account of sampling strategy | 6 | Die Analyse erfolgte auf Basis einer für die deutsche Bevölkerung bezüglich Alter und Geschlecht repräsentativen Stichprobe von circa vier Millionen Versicherten. |
|  |  | (*e*) Describe any sensitivity analyses | - | Es wurden keine Sensitivitätsanalysen durchgeführt. |
| Results | | | | |
| Participants | 13* | (a) Report numbers of individuals at each stage of study—eg numbers potentially eligible, examined for eligibility, confirmed eligible, included in the study, completing follow-up, and analysed | 8 | Im Kalenderjahr 2018 betrug die Anzahl der gesetzlich Versicherten in der zugrundeliegenden Stichprobe 4.197.268. |
|  |  | (b) Give reasons for non-participation at each stage | 8 | Davon waren 3.988.695 durchgehend oder bis zum Tod versichert. 188.440 Patienten wiesen mindestens eine der betrachteten IMID-Diagnosen auf (4,7%). |
|  |  | (c) Consider use of a flow diagram | 8 | Siehe Abbildung 1. |
| Descriptive data | 14* | (a) Give characteristics of study participants (eg demographic, clinical, social) and information on exposures and potential confounders | 8 | Patienten mit IMID waren zu 57,6% weiblich und im Mittel 60,8 Jahre alt (Standardabweichung (SD): 17,0). Der Großteil der Patienten mit IMID war zwischen 51 und 80 Jahren alt, mit einem Altersgipfel zwischen 61 und 70 Jahren (Abbildung 2). |
|  |  | (b) Indicate number of participants with missing data for each variable of interest | - | Nicht vorhanden (vgl. Punkt 12c). |
|  |  | ~~(c)~~ *~~Cohort study~~*~~—Summarise follow-up time (eg, average and total amount)~~ |  |  |
| Outcome data | 15* | *~~Cohort study~~*~~—Report numbers of outcome events or summary measures over time~~ |  |  |
|  |  | *~~Case-control study—~~*~~Report numbers in each exposure category, or summary measures of exposure~~ |  |  |
|  |  | *Cross-sectional study—*Report numbers of outcome events or summary measures | 8-9 | 12-Monats-Prävalenz für IMID  Auftreten von IMID-Kombinationen  Häufigkeit von Hospitalisierungen  Häufigkeit von Facharztkontakten |
| Main results | 16 | (*a*) Give unadjusted estimates and, if applicable, confounder-adjusted estimates and their precision (eg, 95% confidence interval). Make clear which confounders were adjusted for and why they were included | 8-9 | Die 12-Monats-Prävalenz für alle sieben IMID betrug 4.724 Patienten pro 100.000 Versicherte (95%-Konfidenzintervall (CI): 4.704-4.745) und war bei Frauen höher als bei Männern (5.369 pro 100.000 vs. 4.063 pro 100.000). […]  Mit Ausnahme der Psoriasis Arthritis lag die beobachtete Häufigkeit mindestens einer weiteren innerhalb des Studienzeitraums dokumentierten IMID in den betrachteten Krankheitsentitäten zwischen 16,9% (Psoriasis) und 27,5% (Spondylitis ankylosans) (Tabelle 1). […]  Patienten mit IMID wiesen mit einem Anteil von 28,6% im Vergleich zur Referenzpopulation (19,5%) 1,5-mal häufiger mindestens eine Hospitalisierung auf (Tabelle 2). […]  Unabhängig von Krankheitsentität und Facharztdisziplin nahm ein höherer Anteil an Patienten mit IMID ambulante Facharztbehandlungen in Anspruch im Vergleich zur jeweiligen Referenzpopulation (Tabelle 3). |
|  |  | (*b*) Report category boundaries when continuous variables were categorized | - | Es wurden keine Kategoriegrenzen verwendet. |
|  |  | (*c*) If relevant, consider translating estimates of relative risk into absolute risk for a meaningful time period | - | Es sind ausschließlich absolute Werte in Bezug auf das Kalenderjahr 2018 dokumentiert. |

Continued on next page

| Other analyses | 17 | Report other analyses done—eg analyses of subgroups and interactions, and sensitivity analyses | - | Nicht vorhanden. |
| --- | --- | --- | --- | --- |
| Discussion | | | | |
| Key results | 18 | Summarise key results with reference to study objectives | 11-12 | Bisherige Forschungsarbeiten zu Epidemiologie und Versorgungssituation konzentrierten sich zumeist auf einzelne IMID. […] Die vorliegende Studie widmete sich daher zum einen der Frage wie häufig ein breites Spektrum verschiedener IMID in der Versorgungsrealität auftreten und wie häufig multiple IMID-Diagnosen beobachtet werden können, und zum anderen in welchem Ausmaß verschiedene spezifische Fachdisziplinen an der Versorgung beteiligt sind. […] Im Vergleich zur Referenzpopulation wiesen Patienten mit IMID zu höheren Anteilen Hospitalisierungen sowie Inanspruchnahmen des Allgemeinmediziners auf, was auf eine höhere Morbidität innerhalb der Studienpopulation hindeutet. […] Insgesamt erscheint der Anteil der Patienten mit IMID, die während des Beobachtungszeitraums keine der betrachteten Fachdisziplinen aufsuchten, mit über 45% jedoch eher hoch. […] |
| Limitations | 19 | Discuss limitations of the study, taking into account sources of potential bias or imprecision. Discuss both direction and magnitude of any potential bias | 13 | Aufgrund des Querschnittdesigns waren Analysen zu Krankheitsverlauf und kausaler Zusammenhänge wie beispielsweise der Einfluss des Krankheitsschweregrades einer primären IMID auf die Entwicklungsrate weiterer IMID nicht möglich. In der zugrundliegenden Datenbasis waren zudem weder Informationen zum Krankheitsschweregrad enthalten, noch ließ sich bestimmen, ob Facharztkontakte tatsächlich zur Behandlung der betrachteten IMID vorgenommen wurden. |
| Interpretation | 20 | Give a cautious overall interpretation of results considering objectives, limitations, multiplicity of analyses, results from similar studies, and other relevant evidence | 14 | Diese Studie bietet unter Verwendung von aktuellen Real-World-Daten eine umfassende, disziplinübergreifende Betrachtung von Epidemiologie und Versorgungssituation von IMID in Deutschland. Die Studienergebnisse deuten auf eine hohe Morbidität sowie einen erhöhten multidisziplinären Versorgungsbedarf bei betroffenen Patienten hin. |
| Generalisability | 21 | Discuss the generalisability (external validity) of the study results | 14 | Um irreversible Organschädigungen bei IMID zu vermeiden, sind eine frühzeitige Diagnosestellung sowie eine optimale Therapiesteuerung mit bedarfsgerechter interdisziplinärer Abstimmung von zentraler Bedeutung. Es werden jedoch weitere Studien basierend auf Längsschnittanalysen benötigt, um Erkenntnisse bezüglich Krankheits- und Therapieverlauf bei Patienten mit IMID für das deutsche Versorgungssetting zu gewinnen. |
| Other information | |  | | |
| Funding | 22 | Give the source of funding and the role of the funders for the present study and, if applicable, for the original study on which the present article is based | 15 | Diese Studie wurde durch die Janssen-Cilag GmbH gefördert. |

*Give information separately for cases and controls in case-control studies and, if applicable, for exposed and unexposed groups in cohort and cross-sectional studies.

**Note:** An Explanation and Elaboration article discusses each checklist item and gives methodological background and published examples of transparent reporting. The STROBE checklist is best used in conjunction with this article (freely available on the Web sites of PLoS Medicine at http://www.plosmedicine.org/, Annals of Internal Medicine at http://www.annals.org/, and Epidemiology at http://www.epidem.com/). Information on the STROBE Initiative is available at www.strobe-statement.org.
